# Economic Burden of Fatigue in Inflammatory Bowel Disease

**DOI:** 10.1093/crocol/otad020

**Published:** 2023-04-20

**Authors:** Ashwin N Ananthakrishnan, Raj Desai, Wan-Ju Lee, Jenny Griffith, Naijun Chen, Edward V Loftus

**Affiliations:** Division of Gastroenterology, Massachusetts General Hospital and Harvard medical School, Boston, MA, USA; Department of Pharmaceutical Outcomes & Policy, University of Florida, Gainesville, FL, USA; Heath Economics and Outcomes Research, AbbVie Inc., North Chicago, ILUSA; Heath Economics and Outcomes Research, AbbVie Inc., North Chicago, ILUSA; Heath Economics and Outcomes Research, AbbVie Inc., North Chicago, ILUSA; Division of Gastroenterology and Hepatology, Mayo Clinic College of Medicine and Science, Rochester, MN, USA

**Keywords:** inflammatory bowel disease, Crohn’s disease, ulcerative colitis, fatigue, economic impact

## Abstract

**Background:**

This retrospective study gathered medical/pharmacy claims data on patients with inflammatory bowel disease (IBD) between January 01, 2000 and March 31, 2019 from the IBM MarketScan commercial claims database to assess the real-world impact of fatigue on healthcare costs in patients newly diagnosed with IBD.

**Methods:**

Eligible participants were ≥18 years, newly diagnosed with IBD (≥2 separate claims), and had ≥12 months of continuous database enrollment before and after fatigue diagnosis. The date of fatigue diagnosis was the index date; participants were followed for 12 months post-index. Patients with (cases) or without (controls) fatigue were matched 1:1 by propensity score matching. Patients with evidence of prior IBD diagnosis/treatment, or those with a chronic disease other than IBD wherein fatigue is the primary symptom, were excluded. Healthcare resource utilization (HCRU), including hospitalizations, inpatient and outpatient visits, and associated costs were compared between cases and controls.

**Results:**

Matched IBD cohorts (21 321 cases/21 321 controls) were identified (42% Crohn’s disease [CD] and 58% ulcerative colitis [UC]) with similar baseline characteristics (average age: 46 years; 60% female). Cases versus controls had significantly more all-cause outpatient visits (incidence rate ratio [IRR], 95% confidence intervals [95% CI]: 1.64 [1.61, 1.67], *P* < .001) and all-cause hospitalizations (IRR [95% CI]: 1.92 [1.81, 2.04], *P* < .001); as well as significantly higher all-cause total direct healthcare costs (mean: $24 620 vs. $15 324; *P* < .001). Similar findings were observed for IBD-related outcomes, as well as in CD- and UC-specific subgroups.

**Conclusions:**

Presence of fatigue is associated with an increase in HCRU and total medical costs among patients newly diagnosed with IBD.

## Introduction

Inflammatory bowel disease (IBD), which encompasses Crohn’s disease (CD) and ulcerative colitis (UC), is a chronic illness characterized by relapsing and remitting inflammation of the gastrointestinal (GI) tract that can cause abdominal pain, diarrhea, fever, and weight loss.^1,2^ In addition to the GI symptoms, one of the most commonly reported symptoms of IBD is fatigue, which affects up to 80% of patients with active IBD; 50% of patients with inactive IBD still report substantial fatigue.^2–5^ In a study assessing patient-reported symptoms within the previous week, patients with IBD reported lack of energy as one of the most burdensome symptoms regardless of disease activity (active vs. inactive).^6^ Patient-reported fatigue, along with disease activity, was independently associated with patient-reported health-related quality of life (HRQoL).^7^ Moreover, in a study of employed IBD patients, 51% stated that fatigue was the most common reason for work absences.^2–4^

Patients with IBD have significantly higher healthcare use than those without IBD, including the number of hospitalizations, emergency department (ED) visits, and surgeries.^8^ In 2014 it was estimated that direct and indirect costs of IBD in the United States were approximately $31.6 billion.^9^ While treatment prices make up a significant portion of overall costs, hospital stays and clinic visits also contribute substantially. Likewise, up to 30% of patients with UC, and 70% of patients with CD, will eventually require surgery to manage disease, further contributing to the high financial burden of disease.^10^ Despite the high prevalence of fatigue in this population, its impact on healthcare resource utilization (HCRU) and associated costs in patients with IBD have not been well described in a US population. Thus, this study evaluated all-cause and IBD-related HCRU and direct healthcare costs among newly diagnosed patients with IBD; data are stratified based on presence of fatigue (with or without fatigue).

## Materials and Methods

### Study Design and Participants

This study was a retrospective, nested case–control analysis using medical and pharmacy claims data from the IBM MarketScan commercial claims database. This database contains records of patient enrollment, inpatient and outpatient medical claims, expenditures, and outpatient prescription drug claims for over 150 million beneficiaries nationwide. Data on patients diagnosed with IBD were collected from January 1, 2000 to March 31, 2019. As this was a retrospective study leveraging de-identified healthcare claims, and no identified information was used; thus, ethics committee approval was not required. Participants had to have at least 12 months of continuous enrollment prior to IBD diagnosis. The first fatigue diagnosis date (after IBD diagnosis) was defined as the fatigue index date (T_0_), and participants had to have continuous enrollment with medical and pharmacy benefits coverage for ≥12 months pre- and post-fatigue index date (T_−12_ and T_12_, respectively). A schematic of the study design is shown in Supplementary Figure S1.

Eligible participants were ≥18 years old with diagnoses of IBD (International Classification of Diseases [ICD]-9/10 diagnosis codes for CD 555.x/K50.x or UC 556.x/K51.x) on 2 separate encounters. Patients were considered as CD or UC subtype if ≥80% of ICD-9/10 codes were 555.x/K50.x or 556.x/K51.x, respectively. Otherwise, patients were considered to have unspecified IBD and were excluded. To be considered newly diagnosed, participants had no prior IBD diagnoses or treatments for IBD in the ≥12-month period before the first IBD diagnosis date (IBD index date). From this study cohort of patients newly diagnosed with IBD, cases (patients with IBD with fatigue) were identified using ICD-9/10 diagnosis codes for fatigue (780.7, R53.81, R53.82, and R53.83).^11^ To be included, patients had to have a diagnosis of fatigue after the IBD index date, with no evidence of a fatigue diagnosis within the previous 12 months. The control group consisted of patients with IBD without a diagnosis of fatigue between their IBD index date to the end of follow-up. Incidence density sampling was used to match each case with a pool of controls based on IBD index date (within ±1 month), age (within ±5 years), and sex. Using propensity score matching, controls were matched and assigned the same index date of fatigue as the matched cases (imputed fatigue index date). Participants with a previous diagnosis of fatigue prior to the first IBD diagnosis date were excluded from both cohorts. Similarly, patients diagnosed with other chronic diseases which could be the primary cause of fatigue (ie, cancer, multiple sclerosis, rheumatoid arthritis, liver cirrhosis, fibromyalgia, and HIV) in the 12 months before IBD index date were also excluded.

### Outcomes

All outcomes were assessed in the 12 months after the fatigue index date or imputed fatigue index date for cases and controls (T_0_–T_12_), respectively. The incidence of all-cause and IBD-related hospitalizations and ED or outpatient visits were assessed, as well as incidence of IBD-related surgery based on the number of events observed during the follow-up time (ie, events per patient per year [PPPY]). All-cause and IBD-related direct healthcare costs PPPY were assessed and included medical costs (inpatient + outpatient costs), pharmacy costs, and total costs (medical + pharmacy + miscellaneous). IBD-related direct healthcare costs were defined as any claim that had a diagnosis of IBD, a code for any IBD-related surgeries, or a pharmacy claim for therapies commonly prescribed to treat IBD. Data are presented as the mean total cost in US dollars (2019). All outcomes were stratified by IBD subtype (CD or UC) and by disease severity. Patients with moderate to severe disease were defined as those who used immunomodulators (methotrexate, azathioprine, and 6-mercaptopurine) or advanced therapies such as biologics (ie, adalimumab, certolizumab, golimumab, infliximab, natalizumab, ustekinumab, tofacitinib, or vedolizumab) or tofacitinib, whereas patients with mild disease had not used these medications.

### Statistical Analysis

To balance the comparison groups (cases vs. controls) and minimize potential bias because of confounding, a 1:1 greedy matching algorithm of propensity scores adjusted for baseline characteristics was used. Propensity scores were calculated using multivariable logistic regression model controlling for baseline characteristics, IBD subtype, Charlson Comorbidity Index (CCI) score, treatment history, HCRU (ie, all-cause direct healthcare costs, prior IBD-related inpatient and outpatient visits), and all-cause treatment costs during the 12-month pre-index baseline period. ICD codes for baseline comorbidities and components included in the CCI calculation are listed in Supplementary Table S1. All-cause and IBD-related incidence rate ratios of hospitalizations, ED and outpatient visits, and IBD-related surgery were calculated for patients with fatigue relative to those without fatigue and determined by negative binomial regression. Aggregated total direct healthcare costs, both all-cause and IBD-related, were calculated as the sum of inpatient, outpatient, and pharmacy costs. Total direct healthcare costs were compared between patients with and without fatigue by a generalized linear model with Tweedie distribution.

### Ethical Considerations

This is a retrospective study leveraging de-identified healthcare claims, and no identified information was used; thus, ethics committee approval was not required.

## Results

### Patient Selection

A schematic of patient selection processes is shown in Supplementary Figure S2. Briefly, 747 497 patients newly diagnosed with IBD between January 1, 2000 and March 30, 2019, were identified. Patients without continuous database enrollment for the 12 months pre- and post-index date, those with undefined IBD, and those <18 years of age were excluded. Patients were separated based on presence (*N* = 23 631) or absence (*N* = 89 084) of fatigue. After 1:1 propensity score matching, there were 21 321 patients in each cohort (IBD patients with fatigue or without fatigue).

### Baseline Patient Demographic and Clinical Characteristics

Baseline demographic characteristics between patients with fatigue and those without fatigue are listed in Table 1. Briefly, before matching, the average age was 46 years, with approximately 45% of participants ≥50 years old. More patients with fatigue were female (60% vs. 49%, *P* < .0001) and one-third of patients had aminosalicylate use. Patients with fatigue had higher CCI scores (0.40 ± 0.97 vs. 0.22 ± 0.68, *P* < .0001), greater IBD-related and other medication use, and greater HCRU at baseline. Approximately 41% and 59% of patients with IBD could be further stratified into CD and UC subtypes, respectively. After propensity score matching, baseline demographic and clinical characteristics were similar between cohorts. Among both cohorts, 88% had mild disease and 12% had moderate to severe disease. The average age was 46 years, with 60% of participants in both cohorts being female. The difference in all-cause direct healthcare costs at baseline was numerically smaller between patients with fatigue and those without fatigue after matching (before: $19 645 vs. $10 642; after: $19 831 vs. $18 924, respectively).

**Table 1.** Baseline demographic and clinical characteristics.

Characteristics	Unmatched cohort			Matched cohort (1:1)^a^			
	IBD patients with fatigue (*N* = 23 631)	IBD patients without fatigue (*N* = 89 084)	*P* ^b^	IBD patients with fatigue (*N* = 21 321)	IBD patients without fatigue (*N* = 21 321)	*P* ^b^	Standardized mean difference^c^
**IBD subtype,** n (%)							
Crohn's disease	9952 (42.1)	36 133 (40.6)	<.0001	8922 (41.9)	8967 (42.1)	.6568	------
Ulcerative colitis	13 679 (57.9)	52 951 (59.4)	<.0001	12 399 (58.2)	12 354 (57.9)	.6568	------
**Age (years),** mean ± SD	46.5 (11.6)	45.6 (13.1)	<.0001	46.4 (11.7)	46.5 (11.9)	.2769	−0.1
**Age group (years),** *n* (%)							
18–29	2370 (10.0)	13 339 (15.0)	REF	2182 (10.2)	2156 (10.1)	REF	------
30–39	4054 (17.2)	15 331 (17.2)	.6845	3696 (17.3)	3714 (17.4)	.8171	------
40–49	6185 (26.2)	19 852 (22.3)	<.0001	5539 (26.0)	5516 (25.9)	.7967	------
50–59	7969 (33.7)	26 196 (29.4)	<.0001	7178 (33.7)	7175 (33.7)	.9753	------
60+	3053 (12.9)	14 366 (16.1)	<.0001	2726 (12.8)	2760 (12.9)	.6176	------
**Female**, *n* (%)	14 273 (60.4)	43 719 (49.1)	<.0001	12 774 (59.9)	12 806 (60.1)	.7352	------
**Region**, *n* (%)							
Midwest	5118 (23.1)	15 472 (25.0)	REF	4977 (23.3)	4934 (23.1)	REF	------
Northeast	4086 (18.5)	12 854 (20.7)	<.0001	3865 (18.1)	3944 (18.5)	.3225	------
South	9550 (43.1)	22 663 (36.5)	<.0001	9168 (43.0)	9121 (42.8)	.6382	------
West	3384 (15.3)	11 027 (17.8)	<.0001	3,311 (15.5)	3,322 (15.6)	.8826	------
**Plan type,** *n* (%)							
CDHP	1344 (6.0)	4527 (6.5)	REF	1282 (6.0)	1240 (5.8)	REF	0.08
Comprehensive	1053 (4.7)	2743 (3.9)	.0002	992 (4.7)	977 (4.6)	.7290	------
EPO	320 (1.4)	1,028 (1.5)	.6493	314 (1.5)	342 (1.6)	.2699	------
HDHP	618 (2.8)	2755 (3.9)	<.0001	600 (2.8)	563 (2.6)	.2683	------
HMO	2729 (12.2)	9648 (13.8)	<.0001	2641 (12.4)	2597 (12.2)	.5143	------
POS	2102 (9.4)	5461 (7.8)	<.0001	2006 (9.4)	1984 (9.3)	.7142	------
POS with capitation	270 (1.2)	862 (1.2)	.9412	265 (1.2)	281 (1.3)	.4895	------
PPO	13 924 (62.3)	43 101 (61.5)	.0001	13 221 (62.0)	13 337 (62.6)	.2451	------
**Days to fatigue onset**,^d^ mean ± SD	872.6 (928.3)	855.5 (855.9)	<.0001	860.6 (863.0)	817.4 (817.0)	<.0001	0.05
**Baseline comorbidities**, *n* (%)							
Anemia	3559 (15.1)	8024 (9.0)	<.0001	3285 (15.4)	3305 (15.5)	.7872	------
Nutritional deficiency	60 (0.3)	67 (0.1)	<.0001	55 (0.26)	41 (0.19)	.1488	0.1
Depression	2415 (10.2)	4380 (4.9)	<.0001	2190 (10.3)	2193 (10.3)	.9607	------
Anxiety	2655 (11.2)	5630 (6.3)	<.0001	2424 (11.4)	2425 (11.4)	.9876	------
Sleep disorders	1736 (7.4)	3,062 (3.4)	<.0001	1577 (7.4)	1588 (7.5)	.8356	------
Abdominal pain	7907 (33.5)	18 644 (20.9)	<.0001	7384 (34.6)	7480 (35.1)	.3143	–0.1
Diarrhea	5237 (22.2)	14 332 (16.1)	<.0001	4893 (23.0)	4875 (22.9)	.8355	------
**Charlson Comorbidity Index**, mean ± SD	0.40 (0.97)	0.22 (0.68)	<.0001	0.40 (0.96)	0.40 (0.96)	.6612	------
**Baseline IBD-related medication use**, *n* (%)							
Aminosalicylate (5-ASA)	8449 (35.8)	27 744 (31.1)	<.0001	8014 (37.6)	7959 (37.3)	.5819	0.01
Oral corticosteroids	5952 (25.2)	14 409 (16.2)	<.0001	5586 (26.2)	5,444 (25.5)	.1102	0.02
Biologics	1569 (6.6)	4376 (4.9)	<.0001	1453 (6.8)	1353 (6.4)	.0497	0.02
Immunomodulators	1751 (7.4)	4908 (5.5)	<.0001	1649 (7.7)	1592 (7.5)	.2980	0.01
**Baseline other medication use**, *n* (%)							
Antidepressants	5757 (24.4)	10 566 (11.9)	<.0001	5303 (24.9)	5283 (24.8)	.8059	-----
Antibiotics	1158 (4.9)	2468 (2.8)	<.0001	1073 (5.0)	1066 (5.0)	.8765	-----
Erythropoietin stimulating agents	39 (0.2)	58 (0.1)	.0040	34 (0.2)	32 (0.2)	.8056	-----
**Baseline HCRU and costs (USD)**, mean ± SD							
IBD-related outpatient visits	1.9 (3.1)	1.4 (2.5)	<.0001	1.9 (3.1)	1.9 (3.1)	.1771	0.01
IBD-related inpatient visits	0.16 (0.51)	0.09 (0.36)	<.0001	0.16 (0.51)	0.16 (0.51)	.9614	-----
All-cause direct healthcare costs	19 645 (46 897)	10 642 (29 780)	<.0001	19 831 (43 872)	18 924 (48 833)	.0350	0.02

^a^Eligible participants were matched 1:1 based on baseline characteristics, IBD subtype, individual comorbidities, Quan-Charlson Comorbidity score, treatment history, HCRU, and all-cause treatment costs during the 12-month pre-index baseline period.

^b^ *P*-value for fatigue versus no fatigue cohorts.

^c^A difference of more than 0.2 in the standardized difference between cases and controls was considered to be indicative of imbalance.

^d^Calculated as the number of days from the new IBD diagnosis to the first fatigue diagnosis.

Abbreviations: ASA, aminosalicylate; CDHP, consumer-driven health plan; EPO, exclusive provider organization; HCRU, healthcare resource utilization; HDHP, high-deductible health plan; HMO, health maintenance organization; IBD, inflammatory bowel disease; POS, point of service; PPO, preferred provider organization; REF, reference; SD, standard deviation; USD, United States dollar (2019).

### IBD-Related and All-Cause HCRU Incidence Rates

Incidence rates of PPPY of IBD-related hospitalizations and ED and outpatient visits were significantly higher in patients with fatigue versus those without fatigue (Table 2). The incidence rate of hospitalizations and ED and outpatient visits was 89% (IRR [95% CI]: 1.89 [1.74, 2.05], *P* < .0001), 69% (1.69 [1.54, 1.86], *P* < .0001), and 42% (1.42 [1.37, 1.47], *P* < .0001) higher, respectively, in patients with fatigue compared with those without fatigue; fatigued patients also had higher incidence rates of IBD-related surgery. When stratified by CD and UC subtypes, incidence of HCRU for both patients with and without fatigue were similar to those observed in the whole IBD cohort. The incidence rate of all-cause HRCU showed similar trends (Table 2).

**Table 2.** Incidence and incidence rate ratios of healthcare utilization among matched cohorts with and without fatigue by IBD subtype.

	All-cause HCRU			Disease-specific HCRU		
	Patients with fatigue^a^	Patients without fatigue^a^	IRR (95% CI)^b^	Patients with fatigue^a^	Patients without fatigue^a^	IRR (95% CI)^b^
**IBD (N = 21 321)** ^c^						
Hospitalizations, mean ± SD	0.28 (0.80)	0.14 (0.53)	1.92 (1.81, 2.04)*	0.14 (0.56)	0.08 (0.38)	1.89 (1.74, 2.05)*
ED visits, mean ± SD	0.47 (1.37)	0.27 (1.04)	1.73 (1.65, 1.81)*	0.08 (0.45)	0.05 (0.27)	1.69 (1.54, 1.86)*
Outpatient visits, mean ± SD	17.64 (15.56)	10.76 (11.82)	1.64 (1.61, 1.67)*	2.37 (4.02)	1.67 (3.15)	1.42 (1.37, 1.47)*
IBD surgery, mean ± SD	————	————	————	0.06 (0.30)	0.03 (0.21)	1.91 (1.71, 2.14)*
**CD (N = 17 889)** ^c^						
Hospitalizations, mean ± SD	0.32 (0.89)	0.17 (0.57)	1.90 (1.74, 2.07)*	0.18 (0.62)	0.10 (0.42)	1.76 (1.58, 1.96)*
ED visits, mean ± SD	0.55 (1.56)	0.32 (1.20)	1.71 (1.59, 1.83)*	0.12 (0.59)	0.07 (0.34)	1.67 (1.48, 1.89)*
Outpatient visits, mean ± SD	18.29 (15.62)	11.05 (12.02)	1.66 (1.61, 1.70)*	2.87 (4.55)	2.03 (3.60)	1.42 (1.35, 1.49)*
IBD surgery, mean ± SD	————	————	————	0.07 (0.32)	0.04 (0.23)	1.70 (1.45, 1.98)*
**UC (N = 24 753)** ^c^						
Hospitalizations, mean ± SD	0.24 (0.72)	0.13 (0.51)	1.95 (1.80, 2.12)*	0.12 (0.50)	0.06 (0.34)	2.06 (1.83, 2.31)*
ED visits, mean ± SD	0.41 (1.20)	0.23 (0.90)	1.75 (1.64, 1.86)*	0.05 (0.31)	0.03 (0.21)	1.74 (1.49, 2.03)*
Outpatient visits, mean ± SD	17.17 (15.50)	10.54 (11.67)	1.63 (1.59, 1.67)*	2.01 (3.54)	1.41 (2.76)	1.42 (1.36, 1.49)*
IBD surgery, mean ± SD	————	————	————	0.05 (0.29)	0.03 (0.20)	2.16 (1.83, 2.55)*

^a^Incidence rates are reported as number of events per patient per year.

^b^IRRs are calculated by negative binomial regression of healthcare resource use for patients with fatigue relative to those without fatigue.

^c^Eligible participants were matched 1:1 based on baseline characteristics, IBD subtype, Quan-Charlson Comorbidity score, treatment history, HCRU, and all-cause treatment costs during the 12-month pre-index baseline period.

Abbreviations: CD, Crohn’s disease; CI, confidence interval; ED, emergency department; HCRU, healthcare resource utilization; IBD, inflammatory bowel disease; IRR, incidence rate ratio; SD, standard deviation; UC, ulcerative colitis. *P < .0001 for fatigue versus no fatigue cohorts.

### Direct Healthcare Costs Among Patients With IBD With or Without Fatigue

**Figure 1.**
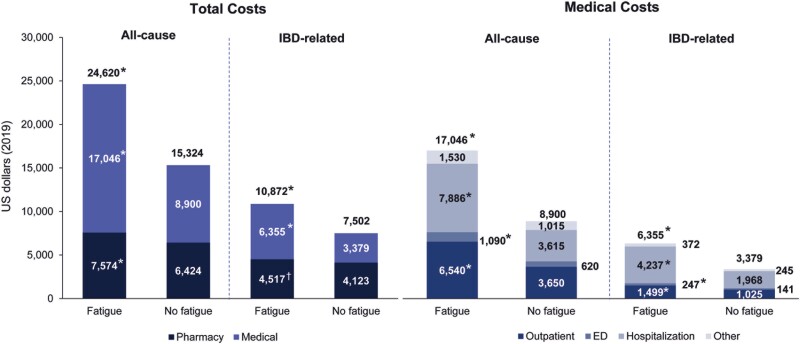
Direct total and medical costs among patients with IBD by presence of fatigue. ED, emergency department; IBD, inflammatory bowel disease. *P < .0001, †*P* = .0003 for fatigue versus no fatigue cohorts.

Overall total IBD-related and all-cause healthcare costs, which included pharmacy and medical costs, were significantly higher in patients with fatigue when compared with those without fatigue (IBD-related: $10 872 vs. $7502, *P* < .0001; all-cause: $24 620 vs. $15 324, *P* < .0001; Figure 1). When stratified by IBD-related medical and pharmacy costs, patients with fatigue had significantly higher costs than patients without fatigue (medical: $6355 vs. $3379, *P* < .0001; pharmacy: $4517 vs. $4123, *P* = .0003); findings for all-cause medical and pharmacy costs were similar. When further broken down by type of medical cost (outpatient or ED visits or hospitalizations; Figure 1), costs were nearly doubled in patients with fatigue, as compared to those without, for IBD-related outpatient and ED visits and hospitalizations (outpatient: $1499 vs. $1025, *P* < .0001; ED: $247 vs. $141, *P* < .0001; hospitalizations: $4237 vs. $1968, *P* < .0001); a similar trend was shown for all-cause medical costs.

### Direct Healthcare Costs Among Patients With CD and UC With or Without Fatigue

**Figure 2.**
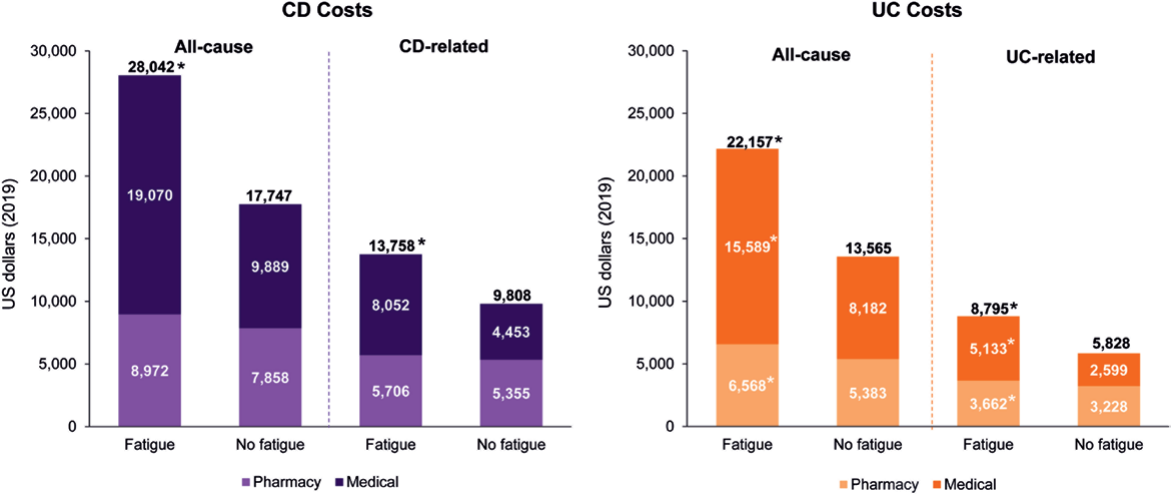
Direct healthcare costs among patients with CD and UC by presence of fatigue. CD, Crohn’s disease; UC, ulcerative colitis. *P < .0001 for fatigue versus no fatigue cohorts.

Patients with CD with fatigue had higher overall total costs compared with patients with CD without fatigue (all-cause: $28 042 vs. $17 747, *P* < .0001; CD-related: $13 758 vs. $9808, *P* < .0001; Figure 2). CD-related medical and pharmacy expenditures were higher in patients with fatigue compared with those without fatigue (medical: $8052 vs. $4453, *P* < .0001; pharmacy: $5706 vs. $5355, *P* < .0001) and similar trends were observed for all-cause medical and pharmacy costs.

In patients with UC, total costs of PPPY were also significantly higher in fatigued patients compared with those without fatigue (UC-related: $8795 vs. $5828, *P* < .0001; all-cause: $22 157 vs. $13 565, *P* < .0001; Figure 2). Patients with fatigue had higher UC-related medical and pharmacy costs than patients without fatigue (medical: $5133 vs. $2599, *P* < .0001; pharmacy: $3662 vs. $3228, *P* < .0001). Similarly, all-cause medical and pharmacy costs were significantly higher in patients with fatigue versus those without fatigue.

### Total Medical Costs Among Patients With IBD With or Without Fatigue by Disease Severity

**Figure 3.**
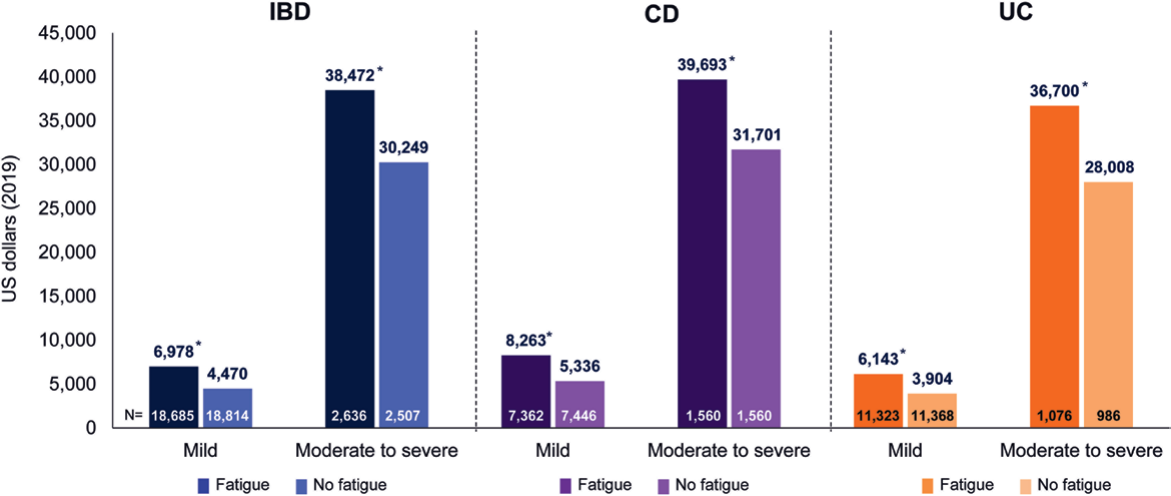
Direct total healthcare costs among patients with IBD, CD, and UC by presence of fatigue and disease severity. CD, Crohn’s disease; IBD, inflammatory bowel disease; UC, ulcerative colitis. *P < .0001 for fatigue versus no fatigue cohorts.

When stratified by disease severity (mild vs. moderate to severe), patients with moderate to severe disease, regardless of the presence or absence of fatigue, had markedly higher total costs compared with patients with mild disease (Figure 3). On average, patients with moderate to severe disease had >5-fold higher total costs compared to those with mild disease. Despite the differences between patients with mild versus moderate to severe disease, the presence of fatigue resulted in consistently higher total medical costs than the absence of fatigue in IBD, CD, and UC populations. Indeed, patients with mild disease and concurrent fatigue had a total healthcare cost that was, on average, 1.6 times higher than in patients with mild disease without fatigue; a similar difference in cost between patients with fatigue versus those without was also observed in patients with moderate to severe disease. When broken down by age group, there were no significant differences between groups for all-cause and IBD-related costs; however, patients with fatigue had numerically higher costs than patients without fatigue (Supplementary Table S2).

## Discussion

The underlying etiology of fatigue in IBD is unclear and likely complex but may stem from active inflammation, anemia, nutritional deficiency, and medication side effects.^3^ While some of these aspects may be treatable, fatigue is persistent in this population. Indeed, a study of patients with IBD showed that, of the 695 patients reporting fatigue at baseline, only 12% had resolved fatigue at the 6-month follow-up and 62% of patients followed to 12 months (*N* = 355) continued to report fatigue.^12^ Fatigue is defined as persistent exhaustion that is disproportionate to the level of exertion, is not alleviated by rest, and results in the inability to participate in activities.^7^ The impact of fatigue can be characterized by 3 main components: Inability or difficulty starting activities, trouble maintaining activities, and cognitive issues such as difficulty concentrating and/or deficits in memory and emotional stability.^3^

The effect of fatigue on HRQoL and ability to participate in daily activities was recently assessed in 1208 patients with IBD in the Swiss IBD Cohort Study.^2^ Compared with controls, IBD patients were significantly more likely to experience fatigue. Fatigue was also associated with higher anxiety and depression scores and lower HRQoL. Moreover, patients with fatigue reported significantly more absences from work, and approximately one-third reported that fatigue significantly impacted their ability to perform daily activities.^2^ A study of US workers showed that those with fatigue cost employers over $136 billion each year due to health-related loss of work productivity. When fatigue occurred with other conditions, there was a 3-fold increase in the proportion of workers with disease-specific health-related loss of work productivity.^9^ There has been little research on the economic impact of fatigue in patients with IBD, thus this study assessed HCRU and direct total healthcare costs in patients newly diagnosed with IBD reporting concurrent fatigue. Patients with IBD experiencing fatigue had a greater number of hospitalizations, outpatient and ED visits, and IBD-related surgeries than patients without fatigue. Similarly, overall total healthcare costs, both IBD-related and all-cause, were significantly higher in patients with fatigue than in those without fatigue. When stratified by IBD subtype, patients with CD or UC experiencing fatigue reported greater HCRU and costs versus those without fatigue. HCRU and total costs remained significantly higher in patients with fatigue versus those without, regardless of IBD subtype or disease severity. These findings are similar to that seen in other chronic diseases greatly affected by fatigue, such as rheumatoid arthritis, wherein the rate of hospitalizations and total office visits increased by up to 83%, and PPPY costs nearly doubled, in patients with fatigue compared to patients without fatigue.^11^

Recommendations for treating fatigue in patients with active IBD may depend on the source of fatigue. For example, if fatigue is secondary to another illness, such as inflammation^3^ or depression,^2^ the treatment goal would be to treat the primary illness. However, up to 50% of patients with inactive disease report persistent fatigue.^3^ Thus, further evaluations for anemia or nutrient deficiencies, psychosomatic or sleep disorders, or other medication use as contributors to persistent fatigue are necessary and may warrant modifications to the treatment regimen.^3,4^ A study in fatigued patients with IBD showed a significant association between persistent fatigue and prior diagnoses of depression, anxiety, or sleep disorders, suggesting that treatment of these ailments may help to resolve fatigue; however, there is limited evidence to support this.^12^ Sleep disturbances have also been correlated with ongoing inflammatory activity even in the absence of GI symptoms^13^ and patients with CD in clinical remission were shown to have a 2-fold increased risk of flare at 6 months when experiencing sleep impairment.^14^ Despite the high prevalence of fatigue in patients with IBD, the effects of pharmacological interventions on fatigue remain uncertain.^6^

There are several tools to measure fatigue that have been validated in CD and UC populations. The Functional Assessment of Chronic Illness Therapy–Fatigue (FACIT-Fatigue) has been validated in patients with IBD and showed high test–retest reliability after 180 days.^15^ The reliability of the Inflammatory Bowel Disease Fatigue (IBD-F) scale and Multidimensional Assessment Fatigue scale have also been assessed in patients with IBD; both questionnaires demonstrated good test–retest reliability after 6 weeks.^1^

The strengths of this study are that it included a large and diverse population of commercially insured individuals in the United States that reflects the real-world economic burden of fatigue in patients with IBD. These findings are poised to highlight and drive further research into the real-world impact of fatigue in patients with chronic illness.

### Limitations

There are several potential limitations of this study. Limited research has been conducted to validate fatigue coding using administrative data, and there are no validated ICD codes for fatigue in the literature. The use of this type of data to identify fatigue may represent more severe cases of fatigue because those cases are more likely to be identified on medical and pharmacy claims than milder cases of fatigue. Also, it is possible that the ICD codes used for fatigue in this study may lead to potential misclassification. In such a case, the differences between patients with and without fatigue, as determined in this study, would likely be an underestimation. While we attempted to adjust outcomes for disease severity and baseline characteristics, it is possible that residual or unmeasured confounders (eg, laboratory results) may still exist in this population. Additionally, despite propensity score matching the costs at baseline were still higher in patients with fatigue suggesting they were a potentially “more severe” group.

## Conclusion

This study demonstrated the significant impact of fatigue on HCRU and cost in patients with IBD regardless of disease severity. These findings pave the way for better recognition of fatigue as an important aspect of disease care in IBD and serve as a call to increase research on the real-world impacts and management of fatigue.

## Supplementary Material

otad020_suppl_Supplementary_Material

## Data Availability

The datasets generated and/or analyzed during the current study are available from the corresponding author upon reasonable request.

## References

[1] Norton C , Czuber-Dochan W, Bassett P, et al. Assessing fatigue in inflammatory bowel disease: comparison of three fatigue scales. Aliment Pharmacol Ther. 2015;42(2):203–211. 25989464 10.1111/apt.13255

[2] Schreiner P , Rossel JB, Biedermann L, et al.; Swiss IBD Cohort Study Group. Fatigue in inflammatory bowel disease and its impact on daily activities. Aliment Pharmacol Ther. 2021;53(1):138–149. 33159475 10.1111/apt.16145

[3] Borren NZ , van der Woude CJ, Ananthakrishnan AN. Fatigue in IBD: epidemiology, pathophysiology and management. Nat Rev Gastroenterol Hepatol. 2019;16(4):247–259. 30531816 10.1038/s41575-018-0091-9

[4] Nocerino A , Nguyen A, Agrawal M, Mone A, Lakhani K, Swaminath A. Fatigue in inflammatory bowel diseases: etiologies and management. Adv Ther. 2020;37(1):97–112. 31760611 10.1007/s12325-019-01151-w PMC6979464

[5] Grimstad T , Norheim KB, Isaksen K, et al. Fatigue in newly diagnosed inflammatory bowel disease. J Crohns Colitis. 2015;9(9):725–730. 25994356 10.1093/ecco-jcc/jjv091

[6] Farrell D , Artom M, Czuber-Dochan W, Jelsness-Jørgensen LP, Norton C, Savage E. Interventions for fatigue in inflammatory bowel disease. Cochrane Database Syst Rev. 2020;4(4):CD012005. 32297974 10.1002/14651858.CD012005.pub2 PMC7161727

[7] Romberg-Camps MJ , Bol Y, Dagnelie PC, et al. Fatigue and health-related quality of life in inflammatory bowel disease: results from a population-based study in the Netherlands: the IBD-South Limburg cohort. Inflamm Bowel Dis. 2010;16(12):2137–2147. 20848468 10.1002/ibd.21285

[8] Terlizzi EP , Dahlhamer JM, Xu F, et al. Health Care Utilization Among U.S. Adults With Inflammatory Bowel Disease, 2015–2016. US Department of Health and Human Services; Centers for Disease Control and Prevention; National Center for Health Statistics; 2021. https://stacks.cdc.gov/view/cdc/100471. 33663650

[9] Ricci JA , Chee E, Lorandeau AL, Berger J. Fatigue in the U.S. workforce: prevalence and implications for lost productive work time. J Occup Environ Med. 2007;49(1):1–10. 17215708 10.1097/01.jom.0000249782.60321.2a

[10] Mehta F. Economic implications of inflammatory bowel disease and its management. Am J Manag Care. 2016;22(3 Suppl):s51-60. 27269903

[11] Strand V , Shah R, Atzinger C, et al. Economic burden of fatigue or morning stiffness among patients with rheumatoid arthritis: a retrospective analysis from real-world data. Curr Med Res Opin. 2020;36(1):161–168. 31433680 10.1080/03007995.2019.1658974

[12] Borren NZ , Long MD, Sandler RS, Ananthakrishnan AN. Longitudinal trajectory of fatigue in patients with inflammatory bowel disease: a prospective study. Inflamm Bowel Dis. 2021;27(11):1740–1746. 33367749 10.1093/ibd/izaa338 PMC8528141

[13] Canakis A , Qazi T. Sleep and Fatigue in IBD: an unrecognized but important extra-intestinal manifestation. Curr Gastroenterol Rep. 2020;22(2):8. 32002666 10.1007/s11894-020-0746-x

[14] Ananthakrishnan AN , Long MD, Martin CF, Sandler RS, Kappelman MD. Sleep disturbance and risk of active disease in patients with Crohn's disease and ulcerative colitis. Clin Gastroenterol Hepatol. 2013;11(8):965–971. 23376797 10.1016/j.cgh.2013.01.021 PMC3659204

[15] Tinsley A , Macklin EA, Korzenik JR, Sands BE. Validation of the functional assessment of chronic illness therapy-fatigue (FACIT-F) in patients with inflammatory bowel disease. Aliment Pharmacol Ther. 2011;34(11–12):1328–1336. 21999576 10.1111/j.1365-2036.2011.04871.x

