# Supplementary material for: Economic Burden of Fatigue in Inflammatory Bowel Disease
Source: Crohns Colitis 360. 2023 Apr 20;5(3):otad020. doi: 10.1093/crocol/otad020 (PMC10470665; doi:10.1093/crocol/otad020)
Supplement: otad020_suppl_Supplementary_Material [file otad020_suppl_supplementary_material.docx]

# Supplementary Materials

### Supplementary Table 1: ICD-9/10 Codes for Baseline Comorbidities and CCI

|  | **ICD-9 diagnosis codes** | **ICD-10 diagnosis codes** |
| --- | --- | --- |
| **Conditions included in CCI calculation** | | |
| Myocardial infarction | 410.x, 411.x | I21.x, I22.x, I25.2 |
| Congestive heart failure | 398, 402, 428 | I09.9, I11.0, I13.0, I13.2, I25.5, I42.0, I42.5-I42.9, I43.x, I50.x, P29.0 |
| Peripheral vascular disease | 440-447, 38.13, 38.14, 38.16, 38.18, 38.43, 38.44, 38.46, 38.48, 38.33, 38.34, 38.36, 38.38, 39.22, 39.29, 39.27, 39.22-39.26, 39.28, 39.29 | I70.x, I71.x, I73.1, I73.8, I73.9, I77.1, I79.0, I79.2, K55.1, K55.8, K55.9, Z95.8, Z95.9 |
| Cerebrovascular disease | 430.x–438.x | G45.x, G46.x, H34.0, I60.x-I69.x |
| Dementia | 290, 290.1, 290.11, 290.12, 290.13, 290.2, 290.21, 290.3, 290.4, 290.41, 290.42, 290.43, 290.8, 290.9, 294.1, 294.11, 331.2 | F00.x-F03.x, F05.1, G30.x, G31.1 |
| Chronic pulmonary disease | 416.8, 416.9, 490, 491, 491.1  491.2, 491.21, 491.22, 491.8, 491.9, 492, 492.8, 493, 493.01, 493.02, 493.1, 493.11, 493.12, 493.2, 493.21, 493.22, 493.81, 493.82, 493.9, 493.91, 493.92, 494, 494.1, 495.x, 496, 500, 501, 502, 503, 504, 505, 506.4  508.1, 508.8 | I27.8, I27.9, J40.x-J47.x, J60.x-J67.x, J68.4, J70.1, J70.3 |
| Rheumatic disease | 710.x, 714.x, 725.x | M05.x, M06.x, M31.5, M32.x-M34.x, M35.1, M35.3, M36.0 |
| Peptic ulcer disease | 531.x-534.x | K25.x-K28.x |
| Mild liver disease | 70.22, 70.23, 70.32, 70.33, 70.44, 70.54, 70.6, 70.9, 570, 571. 571.1, 571.2, 571.3571.4, 571.41, 571.42, 571.49, 571.5, 571.6, 571.8, 571.9, 573.3, 573.4, 573.8, 573.9, V42.7, 456, 456.1, 456.2, 456.21, 572.2, 572.3, 572.4, 572.8 | B18.x, K70.0-K70.3, K70.9, K71.3-K71.5, K71.7, K73.x, K74.x, K76.0, K76.2-K76.4, K76.8, K76.9, Z94.4 |
| Diabetes without chronic complication | 250.0–250.3, 250.7 | E10.0, E10.1, E10.6, E10.8, E10.9, E11.0, E11.1, E11.6, E11.8, E11.9, E12.0, E12.1, E12.6, E12.8, E12.9, E13.0, E13.1, E13.6, E13.8, E13.9, E14.0, E14.1, E14.6, E14.8, E14.9 |
| Diabetes with chronic complication | 250.4–250.6 | E10.2-E10.5, E10.7, E11.2-E11.5, E11.7, E12.2-E12.5, E12.7, E13.2-E13.5, E13.7, E14.2-E14.5, E14.7 |
| Hemiplegia or paraplegia | 334.1, 342.xx | G04.1, G11.4, G80.1, G80.2, G81.x, G82.x, G83.0-G83.4, G83.9 |
| Renal disease | 582.x, 583–583.7, 585.x, 586.x, 588.x | I12.0, I13.1, N03.2-N03.7, N05.2-N05.7, N18.x, N19.x, N25.0, Z49.0-Z49.2, Z94.0, Z99.2 |
| Malignancy | 140.x–172.x, 174.x.–195.8,  200.x–208.x | C00.x-C26.x, C30.x-C34.x, C37.x-C41.x, C43.x, C45.x-C58.x, C60.x-C76.x, C81.x-C85.x, C88.x, C90.x-C97.x |
| Moderate or severe liver disease | 456.0–456.21, 572.2–572.8 | I85.0, I85.9, I86.4, I98.2, K70.4, K71.1, K72.1, K72.9, K76.5, K76.6, K76.7 |
| Metastatic solid tumor | 196.x-199.x | C77.x-C80.x |
| AIDS/HIV | 042.xx, 079.53, v08.xx | B20, R75, Z21 |
| **Comorbidities** | | |
| Anemia | 280, 281 | D500.x, D508.x, D509.x, D51.x, D52.x, D53.x |
| Nutritional deficiency | 269.9 | E63.8 |
| Depression | 293.83, 296.2x, 296.3x, 300.4x | F34.1, F32.x , F33.x, F06.30, F06.31, F06.32 |
| Anxiety | 293.84, 300.0x, 300.2x, 300.3x, 309.21, 309.81, 313.23 | F064, F4000, F4001, F4002, F4010, F4011, F40210, F40218, F40220, F40228, F40230, F40231, F40232, F40233, F40240, F40241, F40242, F40243, F40248, F40290, F40291, F40298, F408-F411, F413, F418, F419, F42, F430, F4310, F4311, F4312, F488, F489, R452, R453, R454, R455, R456, R457, R4581, R4582, R4583, R4584 |
| Cancer | 140.0-239.9 | C00-C96, C7A-C7B |
| Multiple sclerosis | 340.x | G35.x |
| Rheumatoid arthritis | 714.0, 714.81, 714.81, 714.9 | M05.x, M06.x |
| Liver cirrhosis | 571.x | K74.60 |
| Fibromyalgia | 729.1 | M79.7 |
| AIDS/HIV | 042.xx, 079.53, and v08.xx | B20, R75, Z21 |
| Insomnia | 307.42, 307.41, 327.00, 327.01, 327.02, 327.09, 780.52 | F51.x, G47.x |
| Obstructive Sleep Apnea | 780.51, 780.57, 327.23, 780.53 | G47.30, G47.33, G47.39 |
| Diarrhea | 787.91, 564.5x | K59.1, R19.7 |
| Abdominal pain | 789.0x | R109, R1011, R1012, R1031, R1032, R1033, R1013, R1084, R1010, R102, R1030 |

AIDS, acquired immunodeficiency syndrome; CCI, Charlson Comorbidity Index; HIV, human immunodeficiency virus; ICD: International Classification of Diseases.

### Supplementary Table 2: All-Cause and IBD-Related Total Medical Costs by Age Group and Presence of Fatigue

| **Characteristics** | **All-Cause Costs** | | | **IBD-Related Costs** | | |
| --- | --- | --- | --- | --- | --- | --- |
|  | **IBD patients**  **with fatigue**  **(N=21,321)** | **IBD patients**  **without fatigue**  **(N=21,321)** | ***Mean Diff.*** | **IBD patients**  **with fatigue**  **(N=21,321)** | **IBD patients**  **without fatigue**  **(N=21,321)** | ***Mean Diff.*** |
| **Age group (years)**, mean ± SD  18–29  30–39  40–49  50–59  60+ | $29,383 ± $65,399  $20,804 ± $44,847  $21,821 ± $49,433  $25,453 ± $55,881  $29,477 ± $57,286 | $20,984 ± $53,323  $15,112 ± $41,452  $13,764 ± $30,207  $14,783 ± $37,257  $15,708 ± $36,345 | $8398  $5691  $8056  $10,669  $13,768 | $19,175 ± $45,268  $11,391 ± $26,267  $9,976 ± $30,429  $9,445 ± $34,789  $9,099 ± $30,874 | $14,751 ± $35,866  $8,950 ± $22,521  $7,117 ± $20,545  $5,825 ± $21,487  $5,015 ± $18,714 | $4425  $2441  $2859  $3620  $4084 |

IBD, inflammatory bowel disease; SD, standard deviation.

### Supplementary Figure 1: Study design

^
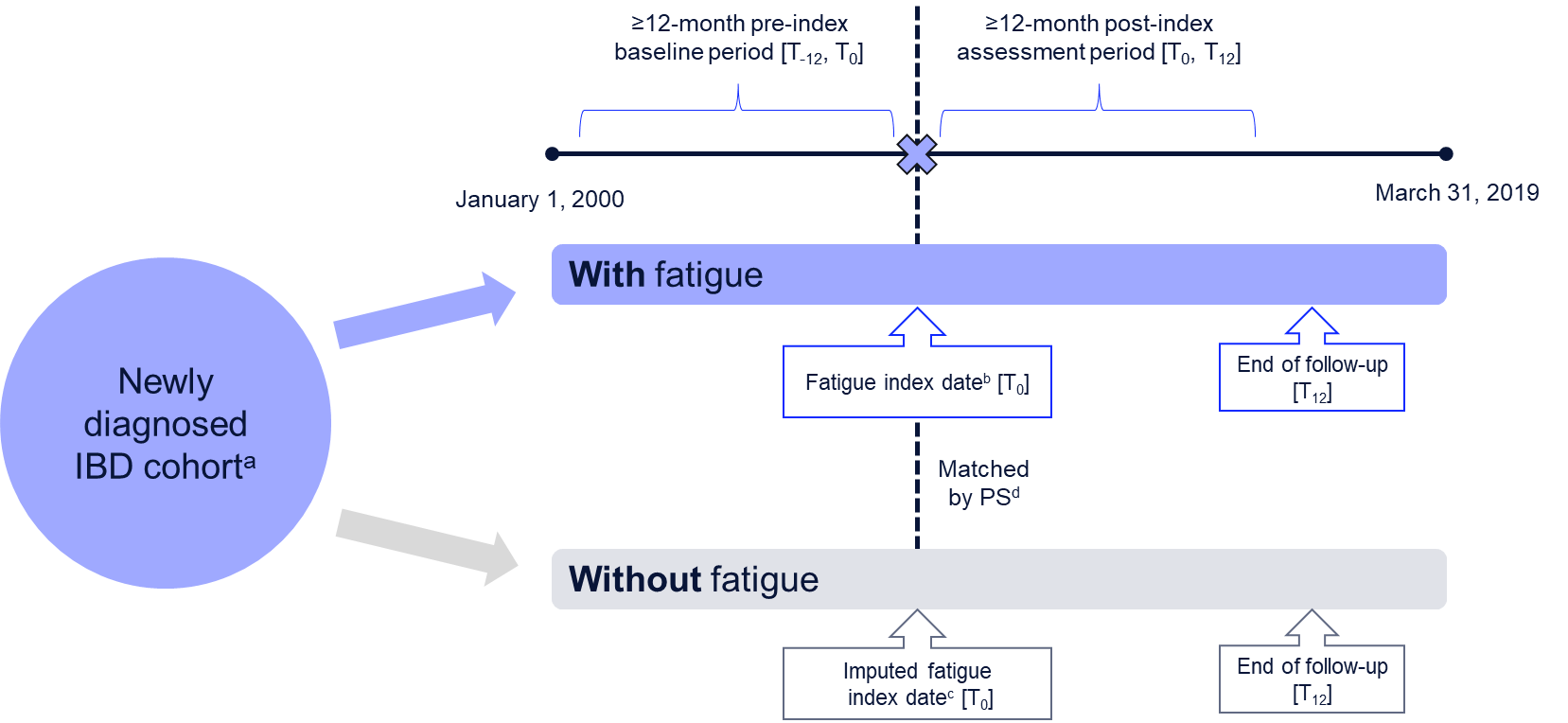
^

^a^Patients had no prior diagnosis of IBD during the 12-month lookback period. ^b^The fatigue index date corresponds to the first claim with a diagnosis of fatigue after the IBD diagnosis date. ^c^Using propensity score matching, controls were matched and assigned the same index date of fatigue as the matched cases (imputed fatigue index date). ^d^Eligible participants were matched 1:1 based on propensity score adjusted for baseline characteristics, IBD subtype, Quan-Charlson Comorbidity score, treatment history, HCRU, and all-cause treatment costs during the 12-month pre-index baseline period. HCRU, healthcare resource utilization; IBD, inflammatory bowel disease; PS, propensity score.

### Supplementary Figure 2: Patient selection and propensity score matching schematic


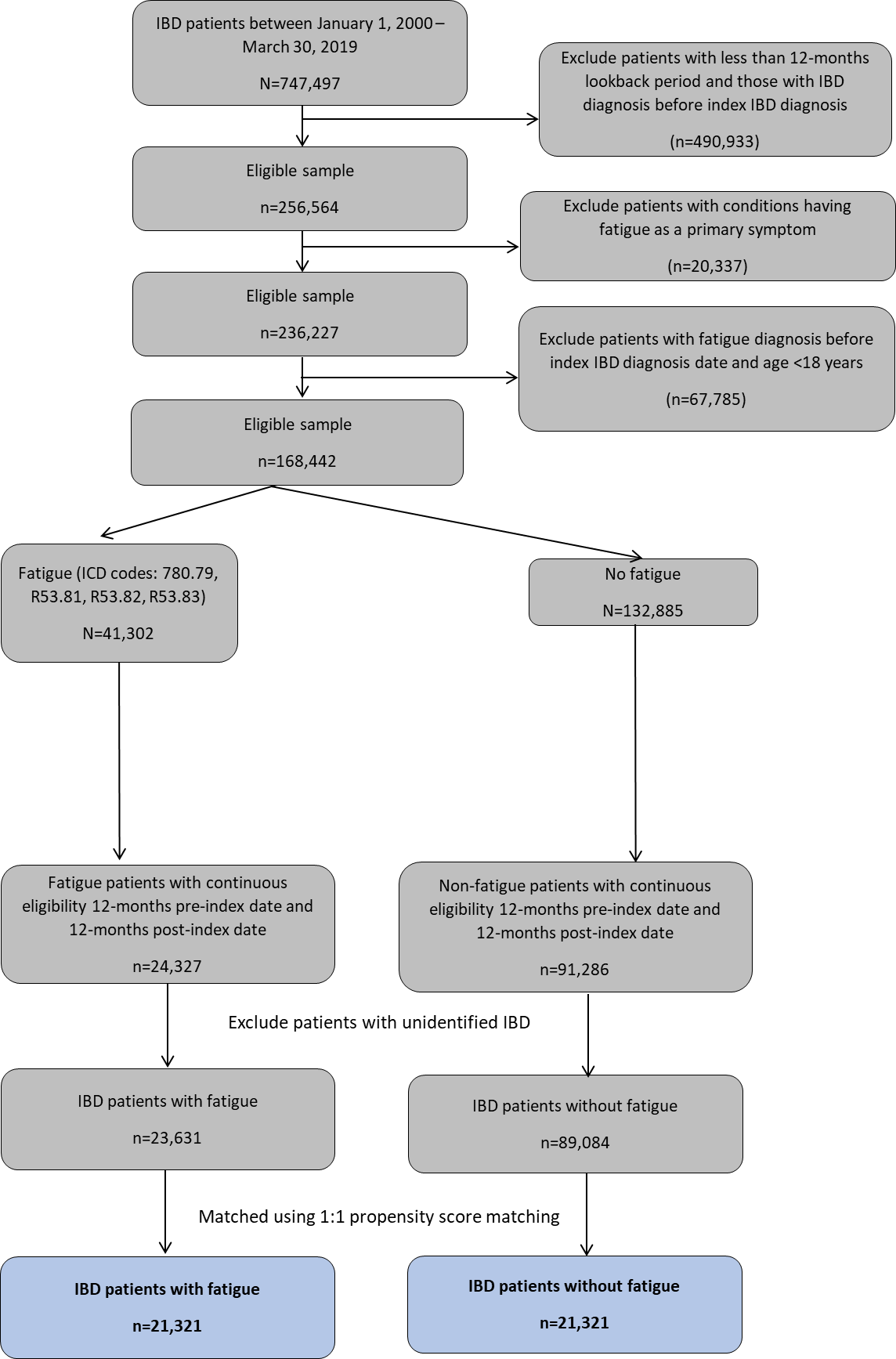


Eligible participants were matched 1:1 based on baseline characteristics, IBD subtype, Quan-Charlson Comorbidity score, treatment history, HCRU, and all-cause treatment costs during the 12-month pre-index baseline period. The 12 months prior to IBD diagnosis is the lookback period. HCRU, healthcare resource utilization; IBD, inflammatory bowel disease; ICD, International Classification of Diseases.
